# A novel *Alteromonas* phage with tail fiber containing six potential iron-binding domains

**DOI:** 10.1128/spectrum.00934-24

**Published:** 2024-11-20

**Authors:** Chen Yu, Meishun Yu, Ruijie Ma, Shuzhen Wei, Min Jin, Nianzhi Jiao, Qiang Zheng, Rui Zhang, Xuejin Feng

**Affiliations:** 1 State Key Laboratory of Marine Environmental Science, College of Ocean and Earth Sciences, Xiamen University, Xiamen, China; 2 Archaeal Biology Center, Synthetic Biology Research Center, Shenzhen Key Laboratory of Marine Microbiome Engineering, Key Laboratory of Marine Microbiome Engineering of Guangdong Higher Education Institutes, Institute for Advanced Study, Shenzhen University, Shenzhen, China; 3 State Key Laboratory Breeding Base of Marine Genetic Resource, Third Institute of Oceanography, Ministry of Natural Resources, Xiamen, China; 4 School of Ocean and Earth Science, Tongji University, Shanghai, China; Universidad Nacional Autonoma de Mexico, Cuernavaca, Mexico

**Keywords:** bacteriophage, *Alteromonas*, interaction, tail structure, iron

## Abstract

Viruses play a vital role in regulating microbial communities, contributing to biogeochemical cycles of carbon, nitrogen, and essential metals. *Alteromona*s is widespread and plays an essential role in marine microbial ecology. However, there is limited knowledge about the interactions of *Alteromonas* and its viruses (alterophages). This study isolated a novel podovirus, vB_AmeP-R22Y (R22Y), which infects *Alteromonas* marina SW-47 (T). Phylogenetic analysis suggested that R22Y represented a novel viral genus within the *Schitoviridae* family. R22Y exhibited a broad host range and a relatively large burst size, exerting an important impact on the adaptability and dynamics of host populations. Two auxiliary metabolic genes, encoding Acyl carrier protein and AAA domain-containing protein, were predicted in R22Y, which may potentially assist in host fatty acid metabolism and VB12 biosynthesis, respectively. Remarkably, the prediction of the R22Y tail fiber structure revealed six conserved histidine residues (HxH motifs) that could potentially bind iron ions, suggesting that alterophages may function as organic iron-binding ligands in the marine environment. Our isolation and characterization of R22Y complements the Trojan Horse hypothesis, proposes the possible role of alterophages for marine iron biogeochemical cycling, and provides new insights into phage-host interactions in the iron-limited ocean.

**IMPORTANCE:**

Iron (Fe), as an essential micronutrient, is often a limiting factor for microbial growth in marine ecosystems. The Trojan Horse hypothesis suggests that iron in the phage tail fibers is recognized by the host’s siderophore-bound iron receptor, enabling the phage to attach and initiate infection. The potential role of phages as iron-binding ligands has significant implications for oceanic trace metal biogeochemistry. In this study, we isolated a new phage R22Y with the potential to bind iron ions, using *Alteromonas*, a major siderophore producer, as the host. The tail fiber structure of R22Y exhibits six conserved HxH motifs, suggesting that each phage could potentially bind up to 36 iron ions. R22Y may contribute to colloidal organically complexed dissolved iron in the marine environment. This finding provides further insights into the Trojan Horse hypothesis, suggesting that alterophages may act as natural iron-binding ligands in the marine environment.

## INTRODUCTION

Viruses are the most abundant biological entities in marine environments, responsible for around 20% of microbial mortality, and thus significantly impacting microbial abundance, diversity, and community structure (1, 2). With each infection, viruses may transfer new genetic information into the organism or progeny viruses, facilitating the evolution of host and virus combinations (3). Viruses exert influence on both the structure and functioning of the entire marine food web, thereby significantly affecting energy flow and material cycling in the ecosystem by infecting bacterioplankton and phytoplankton (4). Despite the nanoscale size, viruses wield a substantial impact on the ocean biogeochemical cycle by metabolically reprogramming host carbon and nitrogen metabolism (5–7).

Iron is an essential nutrient and also one of the primary biological limiting factors in the ocean (8). Studies have indicated that the activity of naturally occurring viral populations is critically important for the recycling of organically complexed iron that supports as much as 90% of primary production in high-nutrient low-chlorophyll (HNLC) surface waters (9). A recent study demonstrated that seven iron ions were present within *Escherichia coli* phage T4, which are coordinated by paired histidine residues (HxH domains) in the tail fiber protein (10). The conserved HxH motifs potential bonding iron ions have been found in the tails of diverse marine phages, including cyanophages and uncultured phages, suggesting that phages serve as organic iron-binding ligands in the marine environment. The Trojan Horse hypothesis suggested that phages utilize iron within the tail fibers to attach to bacterial cells and initiate infection (11). Through the processes of phage infection, assembly, and lysis, host cell materials were remineralized, and progeny phages (containing a significant portion of cellular iron) were released into the environment. Given the dominance of phages among marine viruses and their abundance, the presence or interaction of phages with iron could significantly influence the biogeochemical cycling (12, 13). Thus, the characterization of marine phages and identification of iron-binding conserved protein motifs, especially targeting those capable of infecting dominant bacterial taxon, could continuously interpret viral dark matters from environmental samples and enhance our understanding of the ecological roles of marine phages and their interactions with hosts.

*Alteromonas,* a widely distributed marine genus within the class *Gammaproteobacteria*, thrives in marine environments including coastal waters, open oceans, deep seas, and even hydrothermal areas (14). *Alteromonas* can incorporate the transient nutrients released from phytoplankton and help marine cyanobacteria resist the destruction by hydrogen peroxide (15). It has been found to contribute significantly to the cycling of marine organic carbon and nitrogen (16, 17). In addition, *Alteromonas* exhibited the highest expression of genes involved in iron metabolism in the iron-limited waters of the Southern Ocean. Previous studies have shown that *Alteromonas*, as a siderophore producer, facilitates the availability of iron in global marine communities (18). Therefore, *Alteromonas* has gained more attention as a model strain in microbial oceanographic research due to its vital ecological role. Despite the ecological importance of *Alteromonas*, the characters of *Alteromonas* phages (alterophages) and their possible effects on *Alteromonas* in marine ecosystem and biogeochemical cycling are not well understood.

In this study, we performed a comprehensive analysis of a newly isolated alterophage R22Y to elucidate its morphology characteristics, infection kinetics, genomic features, and evolutionary placement. In addition, we predicted the structure of R22Y tail fiber, revealing potential trimeric iron-binding structures. The investigation contributes to an enhanced comprehension of alterophages genomics and evolution, along with their interactions with hosts, thereby offering novel insights into the potential role of alterophages in iron biogeochemical cycling in the marine environment.

## RESULTS AND DISCUSSION

### The podovirus vB_AmeP-R22Y has a relatively large burst size

**Fig 1:**
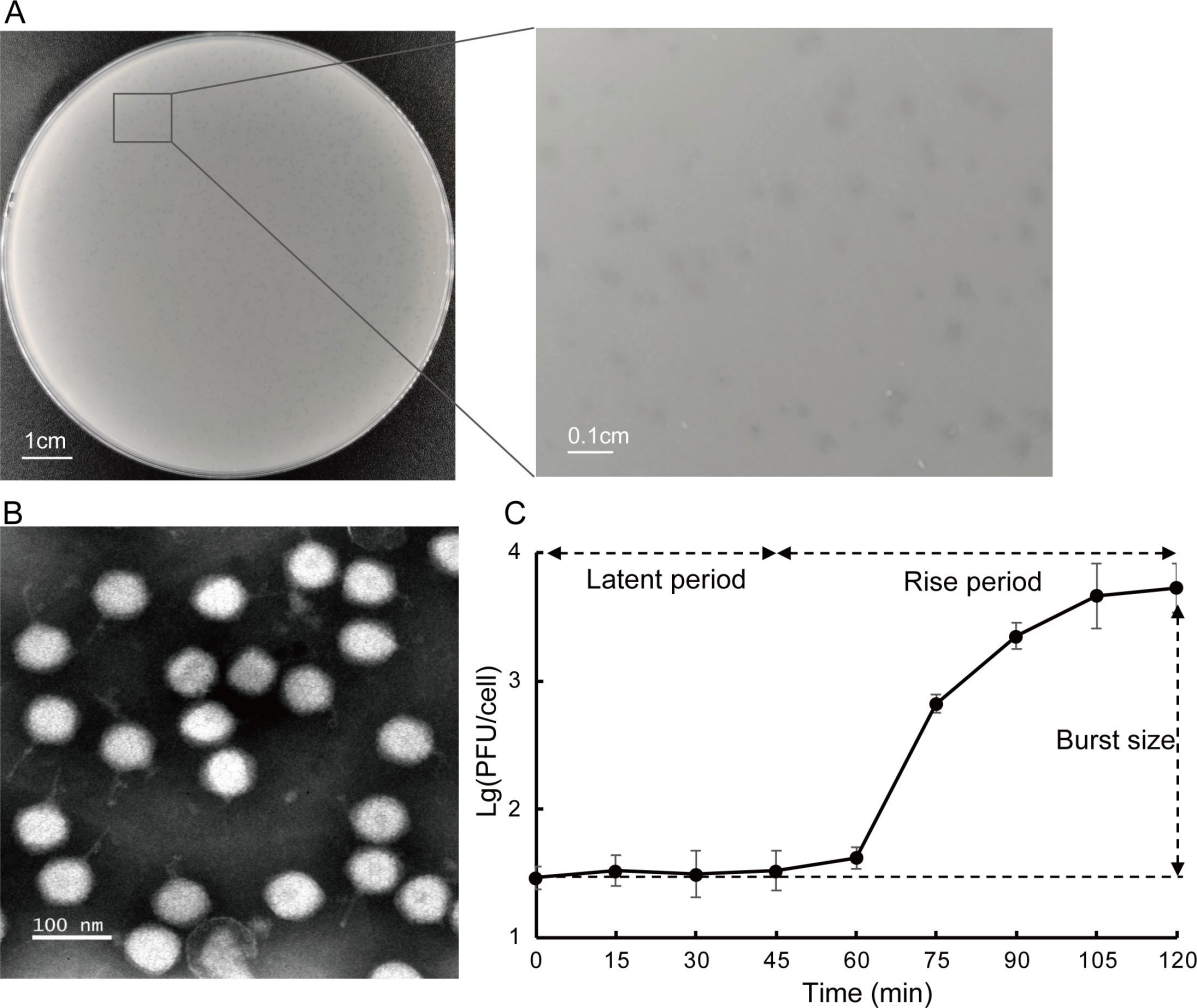
Biological features of R22Y. (**A**) Plaques of R22Y formed on the bacterial lawn of *A. marina* SW-47 (T) after 24 h of incubation. As a reference, the diameter of the culture dish was 90 mm. (**B**) TEM image of R22Y. Scale bar, 100 nm. (**C**) One-step growth curve of phage R22Y and each data point is represented as the mean ± SD derived from three independent replicates.

Bacteriophage vB_AmeP-R22Y (R22Y) was isolated from a seawater sample collected in Dongshan, Fujian, China (117.33°N, 23.57°E), using *Alteromonas marina* SW-47 (T) as a trapping host (19). R22Y forms small round plaques (<1 mm in diameter) with a clear and transparent halo on the host lawn (Fig. 1A). Transmission electron microscopy (TEM) images exhibited that phage R22Y had an isometric head (50 ± 1 nm in diameter) and a short non-contractile tail (60 ± 5 nm) (Fig. 1B). A similar morphotype has been observed for another alterophage vB_AmeP_A5, which has been classified into the *Schitoviridae* family, previously known as the *Podoviridae* family (20). The one-step growth curve revealed that the replication of R22Y within host cells requires approximately 120 min to complete one cycle with a latent period of around 45 min and the rise period of 75 min. The burst size is close to 166 plaque-forming units (PFUs) per cell (Fig. 1C), which is smaller than those of the reported short-tailed alterophages *Schitovirus* vB_AmaP_AD45 P1 to P4 (500 PFUs per cell), *Mareflavirus* ZP6 (210 PFUs per cell), but larger than that of *Autographivirus* vB_AmeP-R8W (88 PFUs per cell) (21–23).

### Overall genomic characteristics of R22Y

**Fig 2:**
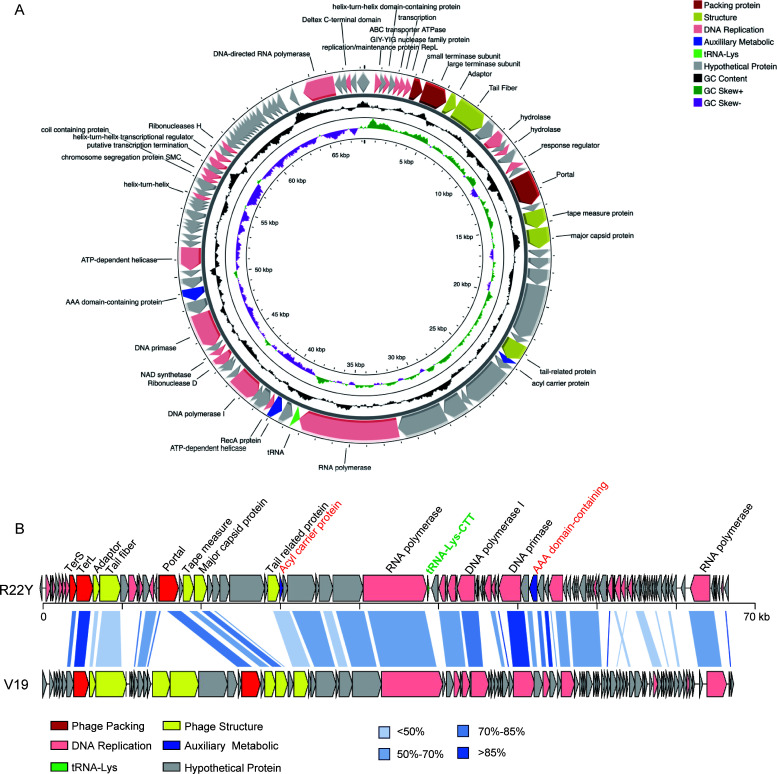
Genomic analysis of phage R22Y. (**A**) Annotated genome of phage R22Y. The predicted functions of proteins are illustrated by different colors of arrows representing genes. (**B**) Full-genome comparison with the phage V19. Homologous proteins are connected using blue shadings of varying transparency to indicate similarity between genes.

Phage R22Y has a double-stranded (ds)DNA with a genome length of 68,251 bp, and an overall G + C content of 37.51%, lower than that of its host (45%). According to PhageTerm, R22Y has a linear genome with direct terminal repeats (DTR) of 335 bp. A total of 99 open reading frames (ORFs) were predicted. Of which, 34 were annotated as functional proteins (Table S1) that can be divided into five categories, such as genome packaging, phage structure, DNA replication, transcription, and auxiliary metabolism (Fig. 2A). The DNA replication module occupied 32.94% of the entire genome and included 24 genes encoding DNA polymerase I, DNA primase, NAD synthetase, and RNA polymerase, etc. An interesting feature of R22Y is the existence of two predicted different RNA polymerase genes (identified as gp36 and gp93), including a very large virion-encapsulated RNA polymerase (vRNAP) consisted of 2,083 amino acids. The large virion RNA polymerase is a representative protein of N4-like phages (24). No genes associated with lysogens (e.g., integrase, repressor) were identified, indicating that R22Y is a lytic phage. Moreover, a tRNA-Lys gene with anticodon CCT was predicted from the R22Y genome. Previous studies have confirmed that phage, in addition to using most of the host’s translation machinery, also uses its own genetic material to supplement it. The presence of tRNA in phages may enable them to gain significant advantages by more effectively translating their proteins, reducing their latency time, and increasing their reproductive rate, that is, to enhance viral virulence (25, 26).

Comparative genomic analysis between R22Y and Alteromonas schitovirus vB_AmeP_PT11–V19 (V19; GenBank OP751378.1) showed significant conservation (amino acid identity >60%, calculated by BLASTp) of key genes involved in the packaging module (TerL and portal protein), the DNA replication module (genes for DNA primase, DNA polymerase, and two RNA polymerases), and structural modules (gene encoding major capsid protein (MCP). Colinear analysis based on amino acid sequences shows regions of median similarity over more than half of the genomes (Fig. 2B). However, the tail fiber gene identified as gp12 in R22Y was only 36% similar to gp4 in V19. Receptor-binding proteins (RBPs), identified as tail fibers or tail spikes, are responsible for recognizing and attaching to the host cell surface, marking the initial step in the infection process (27). The variation in RBPs between R22Y and V19 may result in distinct host-specific recognition and infection mechanisms (28).

### R22Y belongs to a novel genus under *Schitoviridae* family

**Fig 3:**
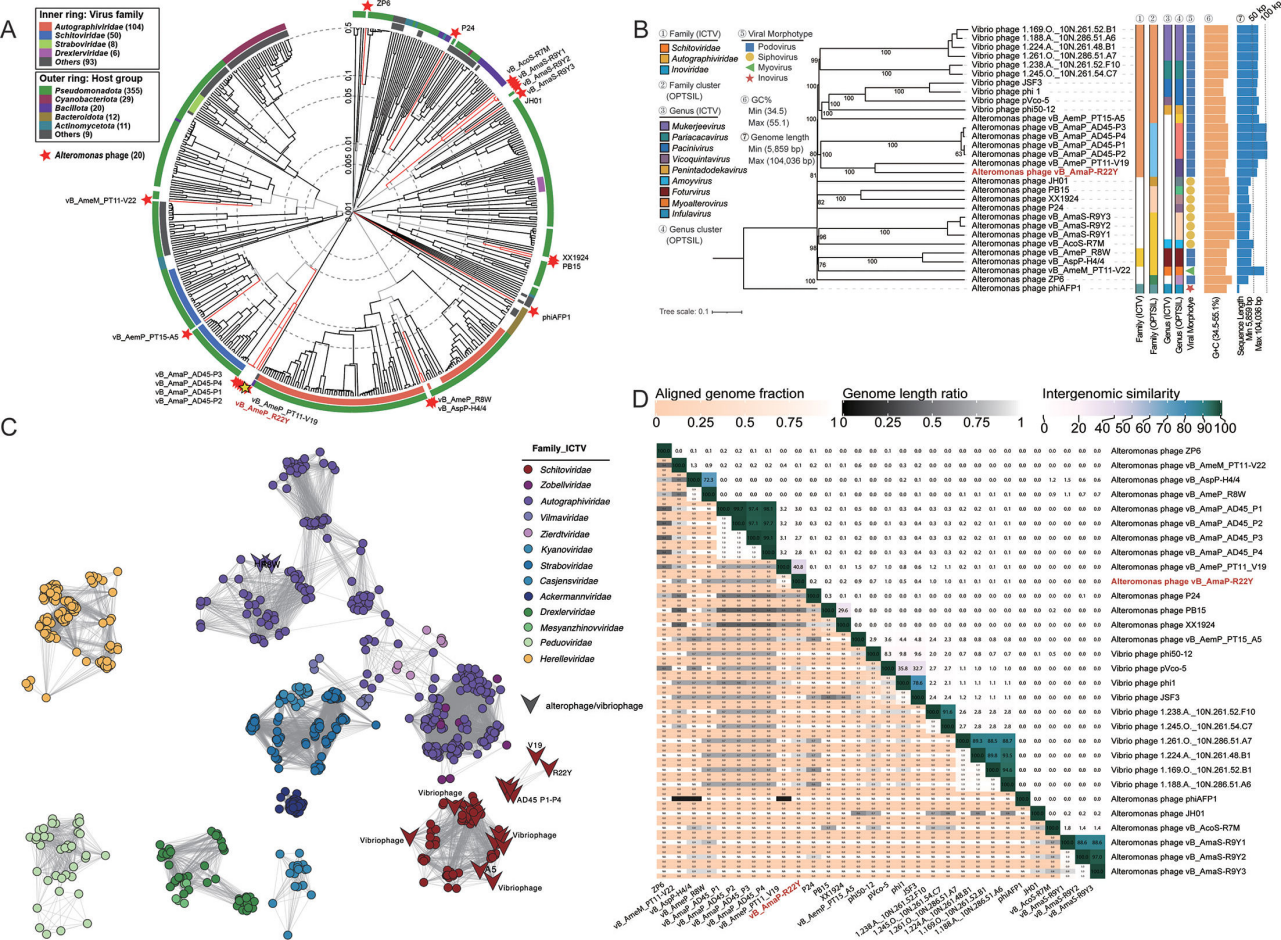
Genomic and taxonomic analyses of R22Y. (**A**) Phylogenetic tree of all 20 *Alteromonas* phages and 422 selected viruses closely related to alterophages. The tree, constructed using VipTree, displays colored rings denoting virus families (inner ring) and host groups (outer ring). (**B**) The GBDP tree of 30 phage genomes reconstructed by VICTOR with the formula D6. (**C**) Protein-based networks of R22Y and its closed phages generated by vConTACT2. Each node represents the phage genomic sequences, with distinct colors indicating their respective host taxonomy. The edges connecting pairs represent the similarity scores between genomes based on shared protein. (**D**) The intergenomic similarity between R22Y and its related phages generated by VIRIDIC.

The proteomic tree of alterophages and other prokaryotic dsDNA viral reference sequences from the Virus-Host DB was generated using Viptree to investigate the taxonomic status of R22Y. Based on alterophages’ branching, 422 phages related to alterophages were selected to elucidate the evolutionary relationships between alterophages and their associated phages (Fig. 3A). The results indicated that R22Y was closely related to alterophages vB_AmaP_AD45 P1 to P4, vB_AmeP_PT11–V19 (V19), vB_AmeP_A5 (A5), and several *Vibrio* phages. This branch of phages belongs to the *Schitoviridae* family, and their hosts belong to the *Pseudomonadota* phylum, including *Alteromonas* and *Vibrio* genus. A total of 19 alterophages and 10 related R22Y family members were further analyzed using the VICTOR server to generate a Genome-BLAST Distance Phylogeny (GBDP) tree (Fig. 3B). Consistently, R22Y and V19 belong to a clade with a long evolutionary distance. According to ICTV taxonomy data, R22Y, V19, P1 to P4, and 10 *Vibrio* phages belong to the *Schitoviridae* family. Meanwhile, phages in this family showed a podophage-like morphology featured by a short tail. Therefore, we proposed that R22Y should be classified into the *Schitoviridae* family.

Furthermore, the results of protein network analysis, in conjunction with ICTV taxonomy, revealed that phages belonging to the *Schitoviridae* family were identified in multiple virus clusters. Alterophages R22Y and V19 exhibited overlaps with P1 to P4, yet showed no correlations with *Vibrio* phages. There was only a single correlation between R22Y and V19 (Fig. 3C). Upon further analysis of intergenomic similarity using the whole-genome length-based algorithm of VIRIDIC, R22Y and its relative phages exhibited intergenomic similarities ranging from 0% to 40.8%, with phage V19 showing the highest similarity (Fig. 3D) (29). Consideration of intergenomic similarity, alignment fractions for each genome pair, and the length ratio between two genomes is crucial for virus classification (30). In accordance with the ICTV classification guidelines, phages can be assigned to a new genus when their nucleotide sequence identity is less than 70% (31). Additionally, all phylogenetic analyses, including terminase large subunit protein (TerL), MCP, DNA polymerase (Fig. S1) showed that R22Y was distinct from all other isolated phages. Overall, R22Y could be identified as a representative of a new genus within *Schitoviridae* family.

### Predicted auxiliary metabolic genes

**Fig 4:**
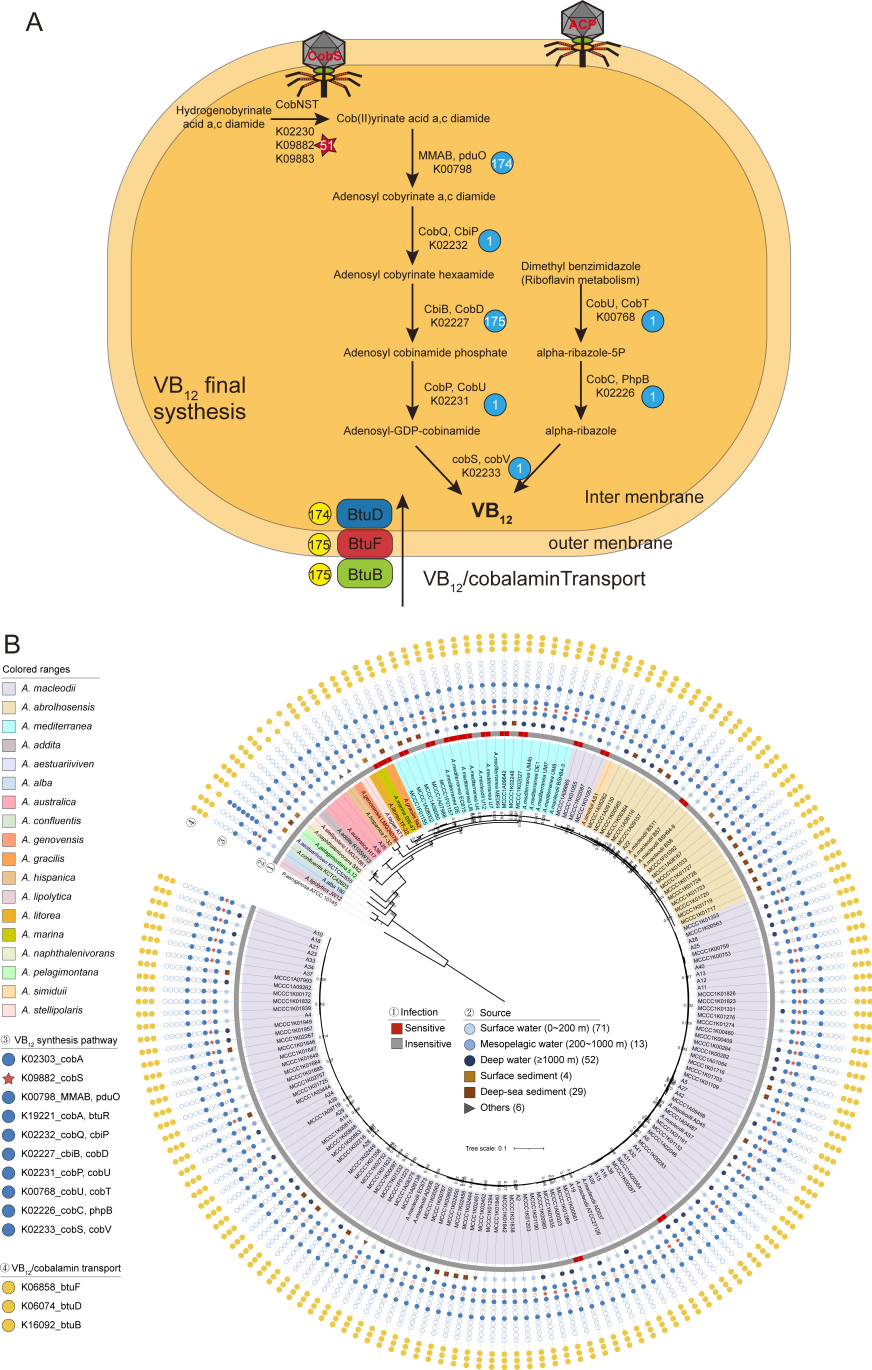
Interactions between phage and host. (**A**) AMGs of R22Y are involved in the metabolism of host bacteria. The flowchart illustrates the involvement of the cobS gene carried by R22Y in the synthesis and transport of host VB12. The related genes, as well as their presence in the 175 strains of host bacteria, are depicted in the figure. (**B**) R22Y lysis profiles of 175 *Alteromonas* strains recovered from various seawater depths. Sensitive and insensitive strains are highlighted in red and gray, respectively. Genes involved in the VB synthesis pathway and transport are shown in blue and yellow, while cobS genes are denoted by pentagrams. Solid indicates the presence of the gene and hollow indicates the absence of the gene. The internal phylogenetic tree for the genus *Alteromonas*, comprising 175 strains, was constructed by concatenating amino acid sequences from 120 bacterial ortholog genes, utilizing GTDB-tk v1.3.0.

AMGs are metabolic genes encoded by phages, originating from hosts, and they regulate host metabolism to enhance viral replication (32). The R22Y genome harbors two predicted AMGs: gp30 (encoding Acyl carrier protein, ACP) and gp53 (encoding AAA domain-containing protein, CobS) (Fig. 4). ACP is a crucial protein that transports intermediates through a series of acyl transfer reactions along the fatty acid biosynthesis pathway (33). It carries the growing acyl chain for bacterial fatty acid synthesis and is targeted by pantothenate antimetabolites, a class of antimicrobial compounds (34). Recently, ACP encoded by phages was identified as an auxiliary metabolic gene involved in fatty acid synthesis. In the *Rhodobacteraceae*, prophages that are most abundant in high latitudes encode ACP, which could play a role in regulating lipid membrane fluidity in polar temperatures (35). Therefore, we hypothesize that ACP in R22Y might provide metabolic benefits to its host, potentially aiding in adaptation to extreme environments such as deep seawaters and deep-sea sediments although further experimental evidence is required to confirm this function.

Another AMG, CobS protein, was also discovered in phages infecting *Cyanobacteria*, *E. coli,* and *Bacteroides fragilis* (36, 37). As one of the subunits of cobaltochelatase, CobS catalyzes the insertion of cobalt into hydrogenobyrinic acid a,c-diamide (HBAD) in the oxygen-dependent (aerobic) cobalamin biosynthetic pathway (B_12_ synthesis) (Fig. 4A) (38). CobS requires ATP and exhibits similarities with magnesium chelatase, and it has been categorized as an AAA+ superfamily protein (39). Consistently, this protein has been annotated as a member of the AAA+ superfamily of proteins in the NR database. Sequences similar to this protein have been annotated in some representative genus of bacteria, such as, *Methanococcoides* and *Caudoviricetes*. Interestingly, CobS is absent in the host of R22Y, but present in 51 tested strains *Alteromonas* (Fig. 4B), suggesting that R22Y may have acquired the gene via horizontal gene transfer from other bacteria. A notable observation is that the cobS gene in R22Y differs from that in *Alteromonas*, indicating distinct evolutionary trajectories (Fig. S2). However, as with ACP, the role of CobS in *Alteromonas* metabolism and its potential impact on phage-host interactions remains speculative and requires further experimental validation.

### The host-specific binding domain

The host range of R22Y was conducted using a large collection of 175 *Alteromonas* strains, including 18 type strains of *Alteromonas* species and 157 non-type *Alteromonas* strains (Table S2). The result showed that R22Y could lyse 5 of the 19 tested species, revealing a broad host spectrum. On a strain level, R22Y could lyse 15 out of 175 tested strains, resulting in a lethal rate of 8.57% (Fig. 4B). Remarkably, a higher portion of susceptible strains was from the *A. mediterranea* species. The tested *Alteromonas* strains were divided into six categories based on their isolation environments, including surface waters, mesopelagic waters, deep-sea waters, surface sediments, deep-sea sediments, and other sources. The corresponding infection rates for each group were found to be 8.5%, 7.7%, 11.5%, 0%, 6.9%, and 0%, respectively (Fig. 4B). Thus, susceptible strains were found across different environmental categories, suggesting a potentially wide geographical distribution of R22Y and its host populations.

**Fig 5:**
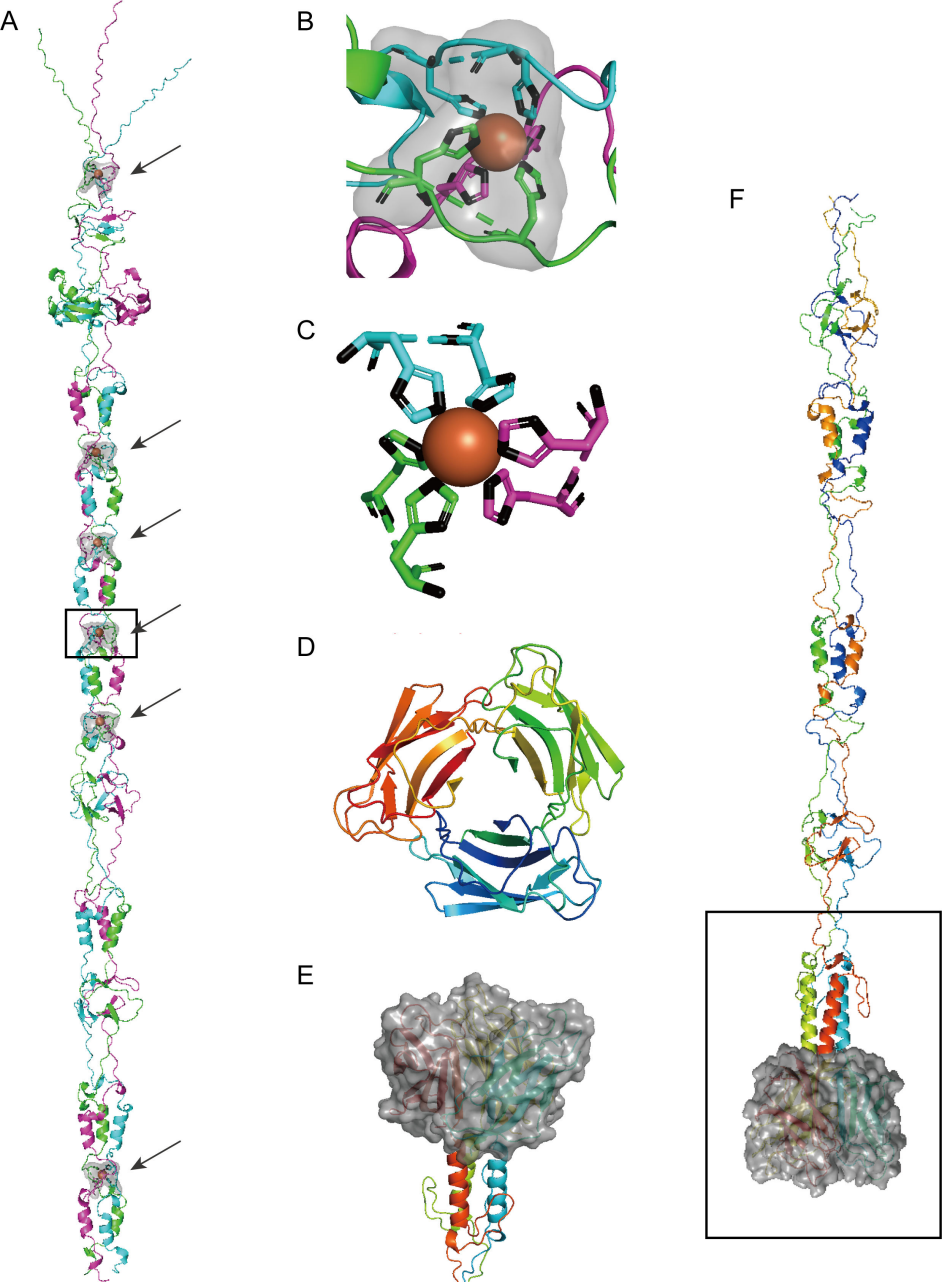
Structures analysis of putative receptor-binding proteins in vB_AmaP-R22Y (R22Y) by using AlphaFold 2.0. (**A**) Ribbon diagrams of RBP trimers (residues1-363) are presented. Chains A, B, and C are colored magenta, green, and cyan, respectively. The six iron ions’ structure are shown in orange. (**B**) Close-up view of the fourth iron ion with coordinated residues in the ion-binding region (black border). (**C**) Top view of iron ion docking to HxH structure. (**D**) Top view of R22Y’s RBP-specific binding region (364–676) to the host surface. (**E**) Close-up view of R22Y’s RBP-specific binding region. (**F**) Ribbon diagrams of RBP trimers (residues 1–676).

Phage recognition and attachment to the specific hosts through encoded RBPs, typically identified as tail fibers or tailspikes, is the first step in infection process and the most critical determinant of host range (40, 41). Furthermore, the host range of phages is determined by the specific structures they use to target bacterial cell (42). The structure of the tail fiber was generated to investigate the unique recognition region of R22Y (Fig. 5). Gp12 in R22Y has been identified as tail fiber that consisted of 676 amino acids. To ensure the accuracy of structural prediction, gp12 was divided into two segments for prediction and analysis, residues 1–363 (Fig. 5A through C, see below) and residues 364–676 (Fig. 5D and E). The results showed that the C-terminal segment (residues 364–676) is a specific receptor-binding domain The RBP-specific binding region of R22Y consists of three β sheets, intricately assembled resembling petals in a flower bud (Fig. 5D). Previous study has characterized new types of alterophages RBPs and identified their essential chaperone for the first time in Alteromonas Myoalterovirus vB_AmeM_PT11-V22 (V22) (43). Subsequently, 13 types of RBPs in published alterophages isolated from 10 different *Alteromonas* species have been identified, highlighting the extensive variability in these proteins (23). Despite this diversity, morphologically distinct alterophages vB_AmeP_PT15-A5, V22, and P24 infected different *Alteromonas* species and strains, while exhibiting a conserved, sequence-homologous, and functionally similar host recognition module. The distinct structures of their tail fibers and chaperones contribute to their differing host range (20). Specifically, the unique structure of the RBP-specific binding region in R22Y may facilitate its recognition of receptors in bacteria from diverse geographical environments. In conclusion, R22Y has a wide host range and a relatively large burst size, exerting an important impact on the adaptability and dynamics of host populations. And it was speculated that R22Y represented an important class of virulent particles that affect the genetics and ecology of *Alteromonas*.

### Potential iron-binding structure

**Fig 6:**
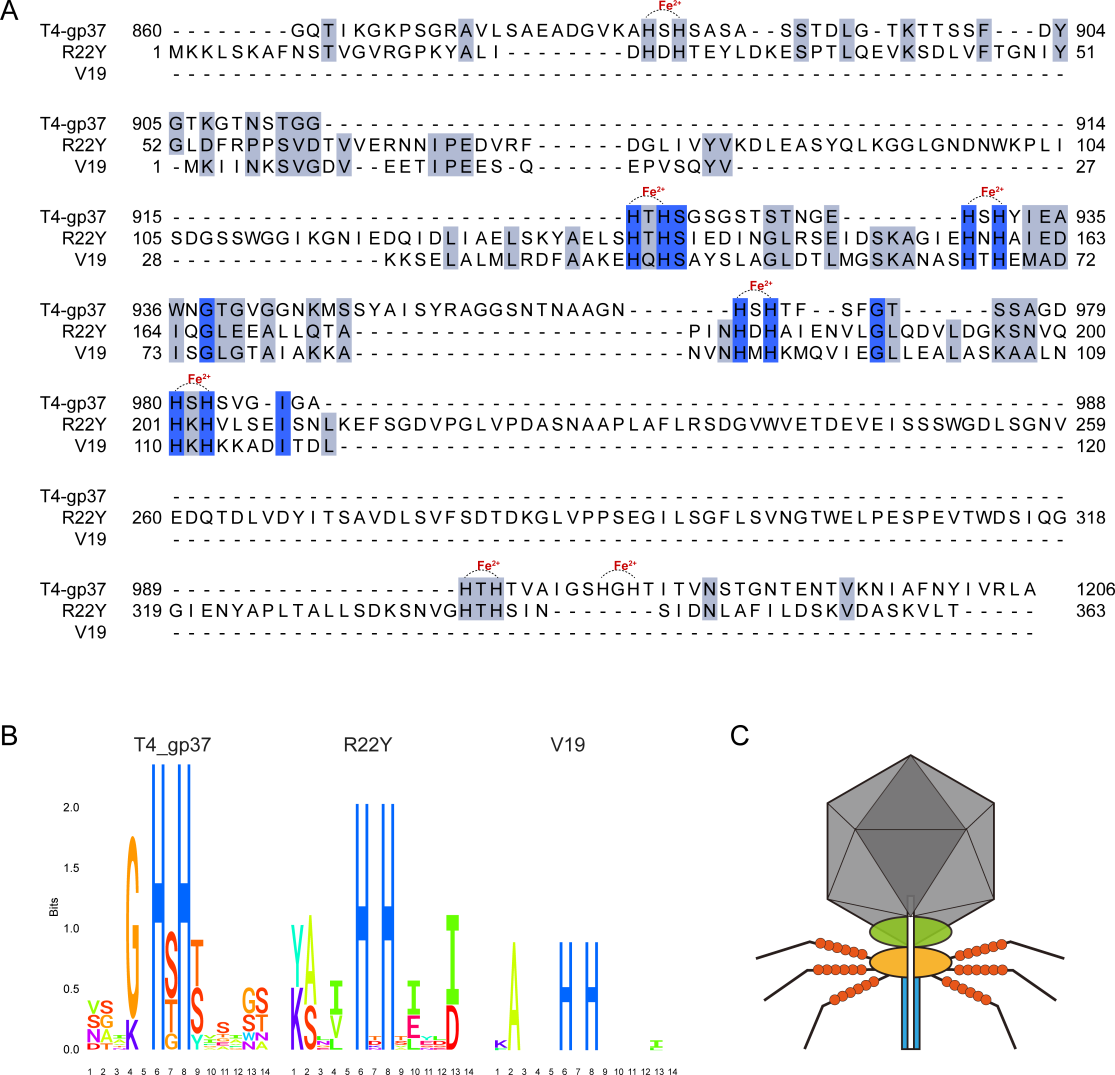
(**A**) Alignment of the segmental domains of the tail fiber gp12 protein (1–363) of R22Y, long tail fiber gp37 protein (811–1206) of phage T4, gp4 protein of phage V19. Identical residues are indicated with dark blue or light blue shadow. HxH motifs are also labeled with Fe^2+^ on top of the alignment. (**B**) HMM logos of the HxH motifs that bind iron ions in phages T4 and conserved HxH motifs identified in the putative tail fiber proteins of R22Y and V19 were created using Skylign. (**C**) Structure of R22Y with six tail fibers binding 36 iron ions (red spheres).

**Fig 7:**
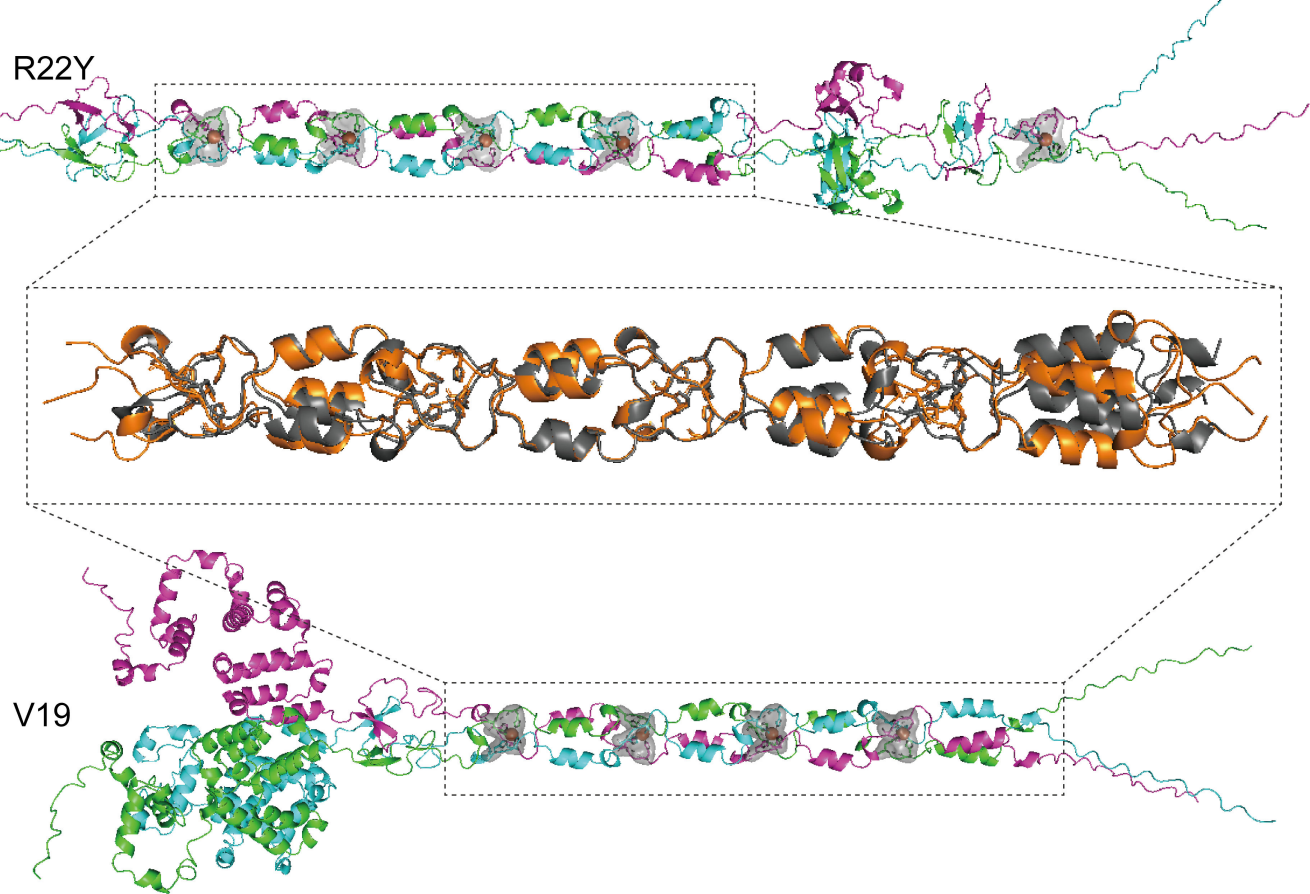
Ribbon diagrams of R22Y as in the tail RBP trimers (tail fiber, residues 1–363, top). In the center, the superposition of R22Y (residues 105–213, orange) and V19 tail fiber (residues 22–116, gray). The tail ﬁbers in the ion-binding site present a conserved domain architecture (RMSD = 1.595). Ribbon diagrams of V19 tail fiber trimers (residues 1–363, bottom).

Recent studies have revealed the histidine residues (His-X-His motifs, HxH motifs) were recurrently presented in the receptor-binding tip of the bacteriophage T4 long tail fiber Gp37 and consistently revealing a binding structure with iron ions, as evidenced by X-ray crystallography (10). The HxH motifs are highly conserved in phage tail fiber or spike, as reported in coliphage Lambda and cyanophages (11, 44). Interestingly, we found six HxH motifs (His-24/His-26, His-135/His-137, His-157/His-158, His-179/His-181, His-201/His-203, and His-338/His-340) at the N-terminal segment of the Gp12 in R22Y. We searched for HxH motifs in the tail fiber protein of all another alterophages and found four in phage V19. By aligning the sequences of the tail fiber gp12 protein (1–363) of R22Y, the long tail fiber gp37 protein (811–1206) of phage T4, and the closest sequence homolog gp4 protein of phage V19, we found that the HxH motifs were highly conserved in alterophages (Fig. 6A and B). HxH motifs of R22Y contributed to the potential binding site of an iron ion in an octahedral coordination sphere (Fig. 5A through C). This observation bears resemblance to the presence of seven iron ions in the HxH motifs of gp37 in the bacteriophage T4. Additionally, we performed a comparative analysis of 3D structure of tail fiber proteins between R22Y and its closest relative V19 (Fig. 7). The result showed that the N-terminal segment of these two proteins exhibits similar structures (RMSD = 1.595). The conserved ion-binding site in R22Y and V19 revealed that alterophages were potential organic iron-binding ligands in the oceans.

It has been suggested that bacteriophages in non-marine systems could infect host by exploiting the siderophore-bound iron receptors on the bacterial cell surface membrane, providing phages with an advantage in the ongoing phage-host evolutionary arms race (11, 45). In contrast to the bacteriophage T4, alterophages possess iron ion-binding sites located at the front part of the tail fiber rather than the tip. This suggested that alterophages might not directly influence host recognition by the presence of iron ions. Instead, they could approach *Alteromonas* through the iron ions in the tail region, thereby increasing the collision probability. We proposed that the continuous presence of iron ions in the tail enhanced the chance of encountering the host, providing an invasion advantage. This perspective supports the Trojan Horse hypothesis, suggesting that iron ions within the phage tail fibers compete with siderophore-bound iron ions for access to the same receptors on the host cell surface (11). *Alteromonas macleodii* has been demonstrated to uptake iron by producing the siderophore petrobactin from weaker iron-ligand complexes, playing a key role in bringing new iron into the ocean (11, 18). Our results showed that six iron ions could be coordinated within each of the six tail fibers of R22Y, potentially amounting to 36 iron ions per phage (Fig. 6C). Thus, iron ions may constitute a stable component of the phage R22Y particle, possibly increasing invasion of host and aiding in the formation of colloidal organically complexed iron, thereby contributing to the marine iron pool.

### Conclusion

This study isolated and characterized a novel *Alteromonas* phage R22Y, which represents a new genus under the *Schitoviridae* family. Our investigation enhanced understanding of the physiology, genetic diversity, and genomic characteristics of alterophages. R22Y exhibited a broad host range and a large burst size, hinting at its significant role in ecological function of *Alteromonas* and the cycling of carbon, nitrogen, and iron. The conserved HxH motifs of tail fiber in alterophages indicated the potential to bind iron ions, suggesting that some alterophages may serve as organic iron-binding ligands in the marine environment. This provides a new window into phage-bacteria interactions in the iron-limited ocean. Further investigations to determine whether R22Y utilizes siderophore-bound iron receptors for host infection in the iron-limited surface ocean could significantly impact our comprehension of marine phage-host interactions.

## MATERIALS AND METHODS

### Phage isolation and purification

The type strain *Alteromonas marina* SW-47 (T) was used as the host for phage isolation. It was grown at 28°C with continuous shaking at a speed of 160 rpm/min in rich organic (RO) medium (1 L artificial seawater supplemented with 0.1% yeast extract, 0.1% peptone, 0.1% sodium acetate, pH 7.4–7.8). The seawater sample for R22Y isolation was collected from the coastal surface water on 14 May 2019, in Dongshan, Fujian, China. The sample was first filtered through a 0.22 µm membrane (Millipore, Massachusetts, USA) to remove bacteria and larger protozoa and then stored in the dark at 4°C. The phage isolation was conducted using the double-layer agar method with certain modifications (46). In brief, 1 mL of filtered seawater was combined with 5 mL of bacteria in the early stages of exponential growth and incubated for 24 h to promote phage enrichment. The 1 mL mixture was re-filtered and added to 1 mL exponentially growing bacteria cultures and 4 mL of molten RO agar medium (0.5% wt/vol agar) for double-layer agar plating. After overnight incubation, clear plaques were isolated, subjected to five additional rounds of purification, and then stored in 1 mL of storage medium (SM) buffer (50 mM Tris-HCl, 0.1 M NaCl, 8 mM MgSO_4_, pH 7.5) at 4°C for further use.

### High-Titer bacteriophage preparation

The 1 L phage lysate was incubated with 2 mg/L of DNase I and RNase A at room temperature for 1 h. Afterward, 1 M NaCl was added and the solution was kept at 4°C for 30 min. Then, centrifuge the mixture at 10,000 × *g* for 10 min at 4°C and filter through a 0.22 µm membrane to remove cellular debris. The filtrate was then treated with 10% (wt/vol) polyethylene glycol 8000 and stored overnight at 4°C to precipitate virions. Then, centrifugation at 10,000 × *g* for 60 min at 4°C resuspends the phage precipitate in 6 mL of SM buffer. And further purified using cesium chloride gradient (1.3, 1.5, and 1.7 g/mL) ultracentrifugation at 200,000 × *g* for 24 h at 4°C. The layer of phage was extracted and dialyzed through 30 kDa super-filters (Millipore, MA, USA) to remove CsCl.

### Transmission electron microscopy

Morphology of the phage was observed by transmission electron microscopy (TEM). In brief, approximately 20 µL of purified and desalted phage suspension should be added to the surface of a copper grid, permitting it to adsorb for 30 min in darkness. Subsequently, the sample should be negatively stained with 1% phosphotungstic acid (pH 7.0) for 1 min and air-dried for 20 min. Finally, images of the phage were examined using a JEM-2100 transmission electron microscope (JEOL, Tokyo, Japan) at 80 kV. The dimensions of phage particles were measured from more than five TEM images by the ImageJ software (47).

### One-step growth curve

Phages were added at a 0.01 multiplicity of infection to 1 mL of exponential growth cultures of the host. This mixture was then incubated in the dark at room temperature for 5 min to allow for phage absorption. To remove any phages not absorbed, the mixture was centrifuged at 10,000 × *g* for 5 min at 4°C. The resulting precipitate was resuspended in 100 mL of RO medium and then incubated at 28°C with continuous shaking at a speed of 180 rpm/min. Phage abundance was counted every 20 min using the double-layer plate method. The burst size for R22Y was calculated by determining the ratio of phages on the growth platform to the initial number of infected host cells (46).

### Host range

The lysis profile of R22Y was determined by using the spot assay method. The tested 175 *Alteromonas* strains, including 18 type strain of species. One milliliter exponentially growing host culture was blended with 5 mL of molten RO agar medium (0.5% wt/vol agar). Subsequently, this mixture was poured onto a solidified RO agar plate (1.5% wt/vol agar) and allowed to stand at room temperature in darkness for 20 min to achieve solidification. The 5 µL phage lysate or SM buffer (as a negative control) was spotted onto the surface of each plate, incubated overnight at 28°C, and subsequently assessed for the presence of clear plaques.

### Genome sequencing and annotation

For phage R22Y DNA extraction, 1 mL of phage stock (1 × 10^12^ PFU/mL) was mixed with 50 µM proteinase K, 20 mM EDTA (pH 8.0), and 0.5% SDS (wt/vol). The mixture was kept at 55°C for 3 h to allow for digestion. Following digestion, the phage DNA was extracted by the phenol-chloroform method and stored at –80°C before sequencing. Phage genomic DNA was sequenced using the Illumina HiSeq 4000 platform, generating a 300 bp paired-end DNA library. The raw data were processed using Soapnuke (v2.0.5) to obtain high-quality clean reads (48). Each sample is independently subjected to *de novo* assembly of clean data using (49) software (v1.1.2, default parameters: --presets meta-large --min-contig-len 300). The termini and packing mechanism of R22Y were determined by PhageTerm (50).

Phage genes were predicted using three different tools: the online GeneMarkS server (51), MetaGeneMark (v3.38) (52), and Prodigal (version 2.6.3) (53). The function of R22Y’s Open reading frames (ORFs) was annotated by BLASTP search algorithms (*e*-value < 0.001), Pfam (*e*-value < 0.01) (54), HHPred (*e*-value < 0.001, cols > 80) (55), Phyre2 (http://www.sbg.bio.ic.ac.uk/phyre2/html/page.cgi?id=inde) (confidence > 40%) (56). Validation of structural gene predictions in the genome was done using VIRFAM (57). The tRNA genes were identified with the tRNA scan-SE program (58). A full-to-full BLASTp approach was used for genome comparisons (bit score of >40) and a customized Java script was employed to create the genome map.

### Phylogenetic analyses

To evaluate the evolutionary relationships between R22Y and other bacteriophages, 5,652 phage genomes were used as reference sequences to construct proteomic trees using VipTree (https://www.genome.jp/viptree), relying on the whole-genome amino acid sequences of phages R22Y and other *Alteromonas* phages (59). R22Y and the relatives performed a network analysis with phages in the ProkaryoticViralRefSeq201-Merged database by vConTACT2 (v0.11.3) based on shared proteins (31). The network was visualized using Cytoscape (V3.9.1) (60). As well, Virus Classification and Tree Building Online Resource (VICTOR, https://ggdc.dsmz.de/victor.php) based on the genome BLAST distance phylogeny (GBDP) method was used to determine R22Y taxonomic position in 29 related phages (61). And the phylogenetic tree was visualized and manipulated using iTOL v6 (62). Additionally, VIRIDIC was obtained using an algorithm that improves over the traditional BLASTN method to compute the intergenomic similarities (30). Moreover, the conserved amino acid sequences of the major capsid protein (MCP), the terminase large subunit (TerL) and DNA polymerase were used to construct marker gene phylogeny. These amino acid sequences were aligned by MUSCLE (v3.8.31), and the maximum-likelihood phylogenetic tree was generated with 1,000 bootstraps by using iqtree (v2.2.0)(63, 64).

### Structure prediction and analysis

The amino acid sequence of the tail fiber gp12 (1–363) of R22Y, the long tail fiber gp37 (811–1,206) of phage T4, and the tail related gp4 of phage V19 were aligned by CLUSTALW (https://www.genome.jp/tools-bin/clustalw) and annotated using ESPript (https://espript.ibcp.fr/ESPript/cgi-bin/ESPript.cgi) (65, 66). HMM logos were created using Skylign for the HxH motifs in the putative tail fiber proteins (67). To investigate the mechanism of recognition and iron-binding, ColabFold v1.5.3 (AlphaFold2 using MMseqs2, https://colab.research.google.com/github/sokrypton/ColabFold/blob/main/AlphaFold2.ipynb) was used to generate 3D structure of the tail fibers (68). The docking of iron ions with HxH motifs was performed by using AutoDock Vina to (version 1.1.2) (69). All structure figures presented in this study were visualized and analyzed using Pymol (PyMOL Molecular Graphics System, version 2.5.5, Schrodinger LLCversion 2.5.5).

## Data Availability

The genome sequence of vB_AmeP-R22Y has been deposited in the GenBank database under the accession number PP212876.1.

## SUPPLEMENTAL MATERIAL

Supplemental figures and tables spectrum.00934-24-s0001.docx Fig. S1 and S2; Tables S1 and S2.

ASM does not own the copyrights to Supplemental Material that may be linked to, or accessed through, an article. The authors have granted ASM a non-exclusive, world-wide license to publish the Supplemental Material files. Please contact the corresponding author directly for reuse.
