# Supplementary material for: A novel Alteromonas phage with tail fiber containing six potential iron-binding domains
Source: Microbiol Spectr. 2024 Nov 20;13(1):e00934-24. doi: 10.1128/spectrum.00934-24 (PMC11705849; doi:10.1128/spectrum.00934-24)
Supplement: Supplemental figures and tables — Fig. S1 and S2; Tables S1 and S2. [file spectrum.00934-24-s0001.docx]

# A novel *Alteromonas* phage with tail fiber containing six potential iron-binding domains

Chen Yu^1,2^, Meishun Yu^1,3^, Ruijie Ma^2^, Shuzhen Wei^4^, Min Jin^3^, Nianzhi Jiao^1^, Qiang Zheng^1^, Rui Zhang^2^*, Xuejin Feng^3^*

^1^State Key Laboratory of Marine Environmental Science, College of Ocean and Earth Sciences, Xiamen University, Xiamen 361102, China

^2^Archaeal Biology Center, Synthetic Biology Research Center, Shenzhen Key Laboratory of Marine Microbiome Engineering, Key Laboratory of Marine Microbiome Engineering of Guangdong Higher Education Institutes, Institute for Advanced Study, Shenzhen University, Shenzhen 518055, China

^3^State Key Laboratory Breeding Base of Marine Genetic Resource, Third Institute of Oceanography, Ministry of Natural Resources, Xiamen 361005, China

^4^School of Ocean and Earth Science, Tongji University, Shanghai 200092, China

**Contents**

**Supplementary Figure S1** Maximum–likelihood phylogenetic trees based on amino acid sequences of phage DNA polymerase, major capsid proteins and TerL.

**Supplementary Figure S2** Maximum–likelihood phylogenetic trees based on amino acid sequences of CobS.

**Supplementary Table S1** Functional annotation of vB_AmeP-R22Y ORFs.

**Supplementary Table S2** Host range of vB_AmeP-R22Y against 175 tested strains of *Alteromonas.*


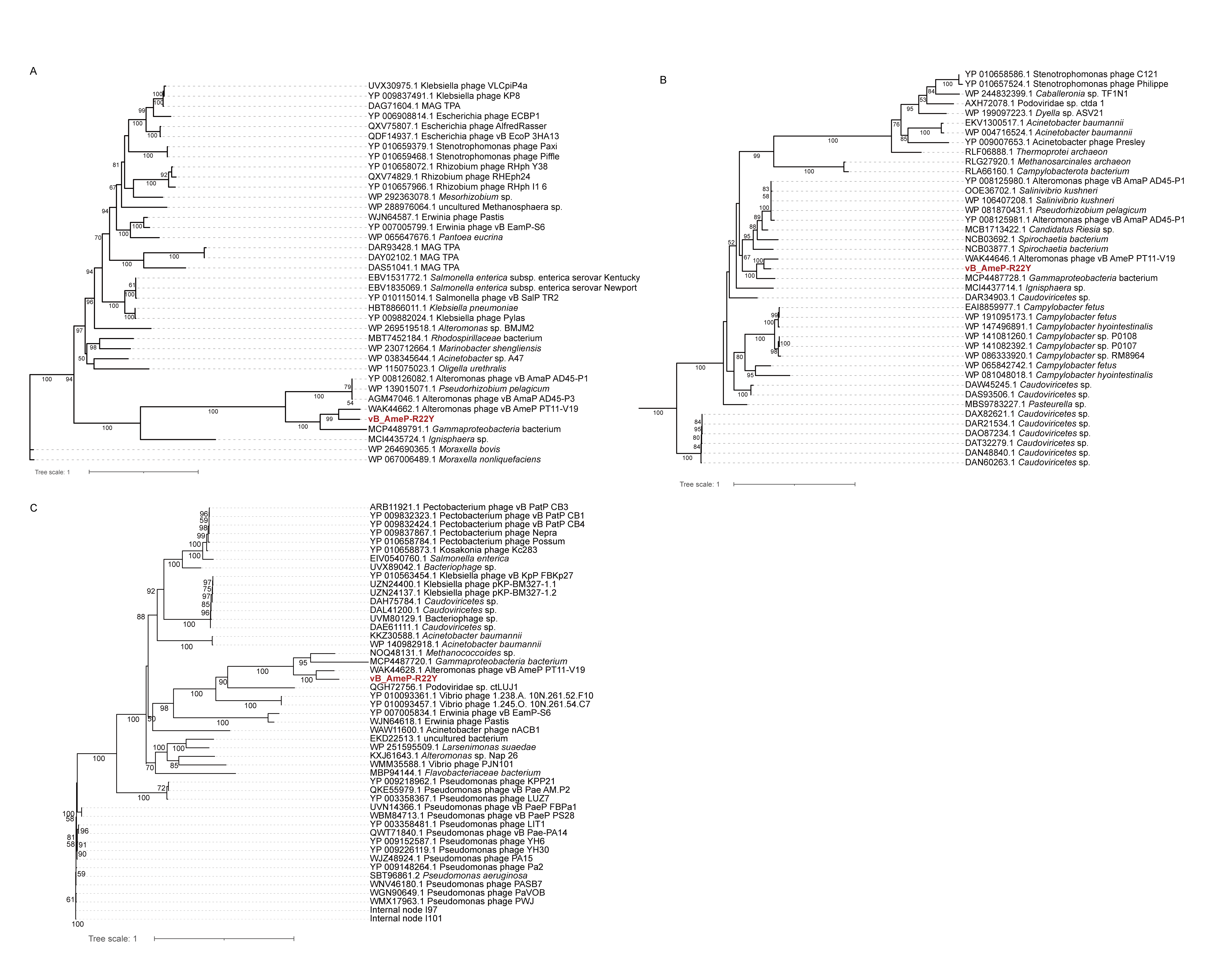


Supplementary Figure S1. Maximum–likelihood phylogenetic trees based on amino acid sequences of phage DNA polymerase (A), major capsid proteins (B), and TerL (C). Bootstrap values are based on 1,000 replicates.


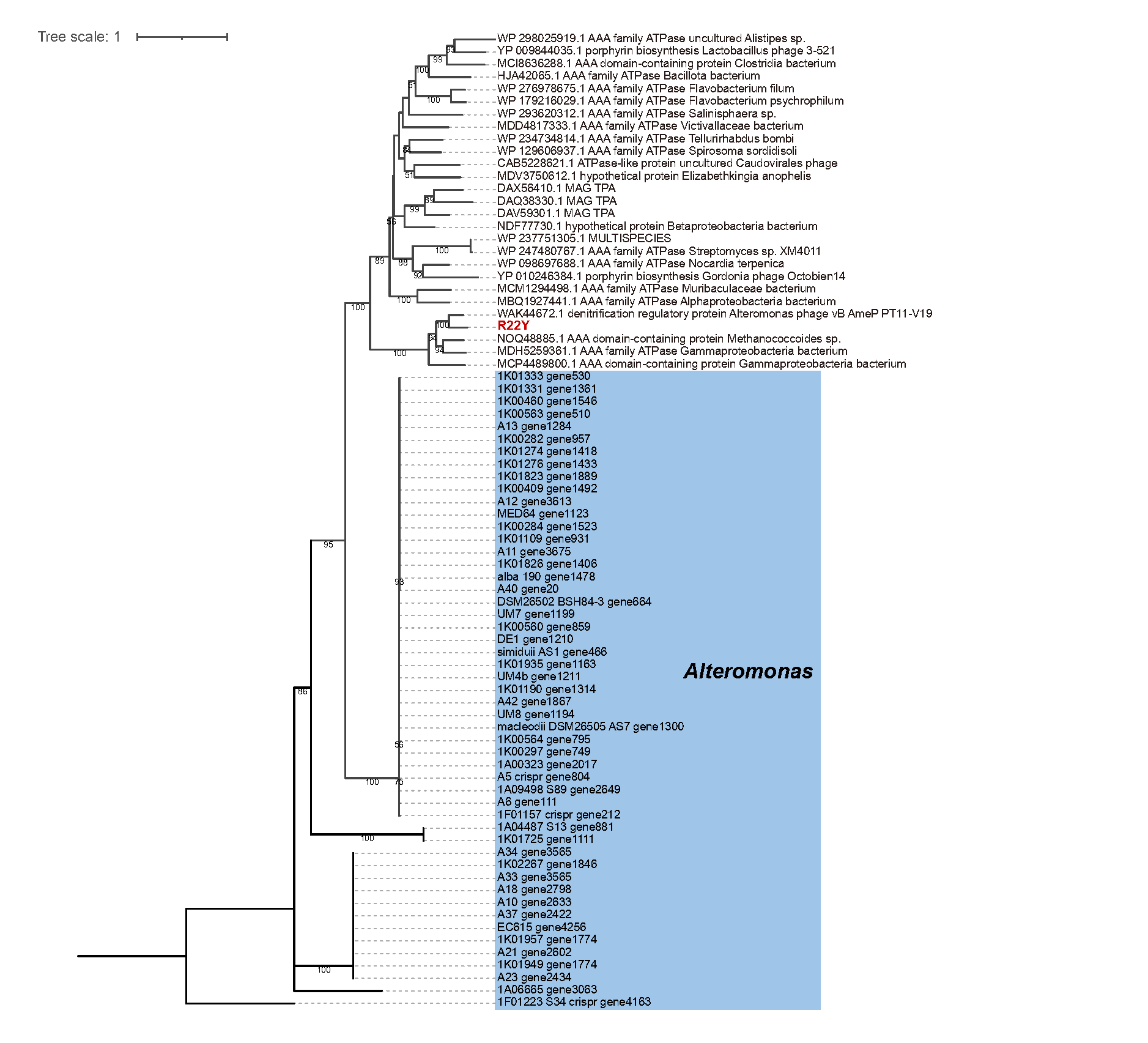
Supplementary Figure S2. Maximum–likelihood phylogenetic trees based on amino acid sequences of CobS.

**Supplementary Table S1. Functional annotation of vB_AmeP-R22Y ORFs.**

| **No.** | **Start** | **Stop** | **Length** | **Strand** | **Putative function** | **Functional categories** |
| --- | --- | --- | --- | --- | --- | --- |
| 2 | 644 | 1066 | 423 | + | Replication protein RepL | replication |
| 4 | 1275 | 1511 | 237 | + | HTH domain-containing protein | transcription |
| 6 | 1893 | 2228 | 336 | + | ABC transporter ATPase | transcription |
| 7 | 2221 | 2541 | 321 | + | Transcription | transcription |
| 8 | 2542 | 2955 | 414 | + | GIY-YIG nuclease family | replication |
| 9 | 2957 | 3673 | 717 | + | Terminase small subunit | packing |
| 10 | 3673 | 5268 | 1596 | + | Terminase large subunit | packing |
| 11 | 5311 | 5967 | 657 | + | Adaptor | structure |
| 12 | 5967 | 7997 | 2031 | + | Tail Fiber | structure |
| 14 | 8832 | 9632 | 801 | + | Hydrolase | lysis |
| 16 | 9868 | 10263 | 396 | + | Hydrolase | lysis |
| 18 | 11307 | 10843 | 462 | - | Response regulator | transcription |
| 20 | 11822 | 13768 | 1947 | + | Portal | packing |
| 22 | 14177 | 15274 | 1098 | + | Tape measure protein | structure |
| 23 | 15292 | 16545 | 1254 | + | Major capsid protein | structure |
| 29 | 22591 | 23787 | 1197 | + | Tail-related protein | structure |
| 30 | 23789 | 24070 | 282 | + | Acyl carrier protein ACP | auxiliary metabolism |
| 36 | 32041 | 38292 | 6252 | + | RNA polymerase | transcription |
| 39 | 40140 | 39478 | 663 | - | Exonuclease | replication |
| 41 | 41152 | 40352 | 801 | - | ATP-dependent helicase | replication and transcription |
| 43 | 43062 | 41281 | 1782 | - | DNA polymerase I | replication |
| 48 | 44674 | 44000 | 675 | - | Ribonuclease D | transcription |
| 49 | 45081 | 44677 | 405 | - | NAD synthetase | Replication |
| 51 | 47587 | 45380 | 2208 | - | DNA primase | replication |
| 53 | 49217 | 48444 | 774 | - | AAA domain-containing | auxiliary metabolism |
| 56 | 51762 | 50350 | 1413 | - | ATP-dependent helicase | replication and transcription |
| 66 | 55061 | 54747 | 315 | - | Helix-turn-helix | transcription |
| 69 | 56170 | 56036 | 135 | - | Coil containing protein | replication |
| 70 | 56721 | 56197 | 525 | - | Chromosome segregation | replication |
| 73 | 57482 | 56901 | 582 | - | Transcription termination | transcription |
| 74 | 57931 | 57479 | 453 | - | HTH transcriptional regulator | transcription |
| 76 | 58631 | 58194 | 438 | - | Ribonucleases H | transcription |
| 93 | 66363 | 64360 | 2004 | - | DNA-directed RNA polymerase | transcription |
| 97 | 67328 | 67044 | 285 | - | Deltex C-terminal domain | transcription |

**Supplementary Table S2 Host range of** **vB_AmeP-R22Y against 175 tested strains of *Alteromonas***

| **Strain** | Taxonomy | Origin | Depth | Infectivity |
| --- | --- | --- | --- | --- |
| SW-47 T | A. marina | Eastern Sea, Korea | Surface | + |
| 9a2 T | A. gracilis | Pacific Ocean | Sediment (6310 m) | + |
| MCCC 1K00564 | A. macleodii | Pacific Ocean | Bathypelagic (5098 m) | + |
| U4 | A. mediterranea | Ionian Sea, Uranian Basin Western of Crete | Bathypelagic (3475 m) | + |
| U7 | A. mediterranea | Ionian Sea, Uranian Basin Western of Crete | Bathypelagic (3500 m) | + |
| U8 | A. mediterranea | Ionian Sea, Uranian Basin Western of Crete | Bathypelagic (3500 m) | + |
| MCCC 1A08002 | A. mediterranea | Southern Atlantic Ocean | Sediment (5610 m) | + |
| MCCC 1A09642 | A. mediterranea | Southern Atlantic Ocean | Bathypelagic (1000 m) | + |
| MCCC 1K02027 | A. mediterranea | South China Sea | Bathypelagic (1700 m) | + |
| MCCC 1K01057 | Alteromonas sp. | South China Sea | Mesopelagic (700 m) | + |
| MCCC 1K01289 | A. macleodii | Western Pacific Ocean | Mesopelagic (100 m) | + |
| MCCC 1F01157 | A. mediterranea | South China Sea | Surface | + |
| EC615 | A. mediterranea | English Channel | Surface | + |
| DSM26498 | A. macleodii | Black Sea Karadag | Surface | + |
| MCCC 1A06665 | A. macleodii | Bali Beach, Indonesia | Beach | + |
| DE T | A. mediterranea | Adriatic Sea, Urania Basin | Bathypelagic (1000 m) | − |
| R10SW13 T | A. addita | Chazhma Bay, Sea of Japan, Pacific Ocean | Surface | − |
| KCTC42603 T | A. confluentis | Jeju Island, South Korea | Surface | − |
| AT1 T | A. tagae | Er-Jen River estuary, Tainan | Surface estuarine | − |
| 5.12 T | A. pelagimontana | Indian Ocean | Sediment (2681 m) | − |
| KCTC52655 T | A. aestuariivivens | Tropical Pacific Ocean | Sediment (tidal-flat) | − |
| SN2 T | A. naphthalenivorans | Southern Atlantic Ocean | Sediment (tidal-flat) | − |
| TF-22 T | A. litorea | Korea, Yellow Sea | Sediment (Intertidal) | − |
| AS1 T | A. simiduii | Er-Jen River estuary, Tainan | Surface estuarine | − |
| JW12 T | A. lipolytica | Arabian Sea, Indian Ocean | Surface | − |
| H 17 T | A. australica | Port Phillip Bay, Tasman Sea, Pacific Ocean | Surface | − |
| 190 T | A. alba | Western Pacific Ocean | Surface | − |
| LMG 24078 T | A. genovensis | Genoa, Italy | Biofilm | − |
| LMG 21861T | A. stellipolaris | Antarctica | Surface | − |
| F-32 T | A. hispanica | Fuente de Piedra, southern Spain | Hypersaline water | − |
| ATCC 27126 T | A. macleodii | Hawaii, Pacific Ocean Oahu | Surface | − |
| DSM26503 | A. macleodii | Black Sea Karadag | Surface | − |
| AD006 | A. macleodii | Port Dickson, Malaysia | Surface | − |
| BS11 | A. macleodii | Black Sea Karadag | Surface | − |
| EC673 | A. macleodii | English Channel | Surface | − |
| DSM26500 | A. macleodii | Black Sea Karadag | Surface | − |
| DSM26505 | A. macleodii | Andaman Sea | N/A | − |
| AD037 | A. macleodii | Port Dickson, Malaysia | Surface | − |
| DSM26502 | A. macleodii | Black Sea Karadag | Surface | − |
| DSM26497 | A. macleodii | Ionian Sea, Uranian Basin Western of Crete | Bathypelagic (3500 m) | − |
| AD45 | A. macleodii | Mediterranean Sea | Surface | − |
| DE1 | A. mediterranea | Adriatic Sea, Urania Basin | Bathypelagic (1000 m) | − |
| UM7 | A. mediterranea | Ionian Sea, Uranian Basin Western of Crete | Bathypelagic (3475 m) | − |
| UM8 | A. mediterranea | Ionian Sea, Uranian Basin Western of Crete | Bathypelagic (3475 m) | − |
| UM4b | A. mediterranea | Ionian Sea, Uranian Basin Western of Crete | Bathypelagic (3455 m) | − |
| MED64 | A. mediterranea | Aegean Sea, Mediterranean | Surface | − |
| A2 | A. macleodii | Atlantic Ocean | Surface | − |
| A4 | A. macleodii | Pacific Ocean | Surface | − |
| A5 | A. macleodii | Western Pacific Ocean | Mesopelagic (200 m) | − |
| A6 | A. macleodii | Philippine Sea | Subsurface (75 m) | − |
| A10 | A. macleodii | South China Sea | Surface | − |
| A11 | A. macleodii | South China Sea | Surface | − |
| A12 | A. macleodii | South China Sea | Subsurface (75 m) | − |
| A13 | A. macleodii | South China Sea | Subsurface (75 m) | − |
| A14 | A. macleodii | South China Sea | Subsurface (75 m) | − |
| A15 | A. macleodii | South China Sea | Subsurface (75 m) | − |
| A16 | A. macleodii | South China Sea | Subsurface (75 m) | − |
| A18 | A. macleodii | South China Sea | Bathypelagic (1000 m) | − |
| A19 | A. macleodii | South China Sea | Bathypelagic (1000 m) | − |
| A20 | A. macleodii | South China Sea | Bathypelagic (1000 m) | − |
| A21 | A. macleodii | South China Sea | Mesopelagic (100 m) | − |
| A22 | A. abrolhosensis | South China Sea | Bathypelagic (1003 m) | − |
| A23 | A. macleodii | South China Sea | Bathypelagic (1000 m) | − |
| A24 | A. macleodii | South China Sea | Bathypelagic (1000 m) | − |
| A25 | A. macleodii | South China Sea | Bathypelagic (4219 m) | − |
| A26 | A. macleodii | South China Sea | Bathypelagic (4054 m) | − |
| A27 | A. macleodii | South China Sea | Bathypelagic (4054 m) | − |
| A28 | A. macleodii | South China Sea | Bathypelagic (3861 m) | − |
| A29 | A. macleodii | South China Sea | Bathypelagic (3861 m) | − |
| A31 | A. macleodii | South China Sea | Bathypelagic (3861 m) | − |
| A32 | A. macleodii | South China Sea | Bathypelagic (2739 m) | − |
| A33 | A. macleodii | South China Sea | Mesopelagic (200 m) | − |
| A35 | A. macleodii | South China Sea | Mesopelagic (200 m) | − |
| A36 | A. australica | South China Sea | Mesopelagic (200 m) | − |
| A37 | A. macleodii | South China Sea | Mesopelagic (200 m) | − |
| A38 | A. macleodii | South China Sea | Surface | − |
| A39 | A. macleodii | South China Sea | Mesopelagic (200 m) | − |
| A40 | A. macleodii | South China Sea | Mesopelagic (200 m) | − |
| A41 | A. macleodii | South China Sea | Mesopelagic (200 m) | − |
| A42 | A. macleodii | Northern Pacific Ocean | Surface | − |
| MCCC 1K01055 | Alteromonas sp. | South China Sea | Surface | − |
| MCCC 1K00172 | A. macleodii | South China Sea | Surface | − |
| MCCC 1K00560 | A. macleodii | Eastern Pacific Ocean | Surface | − |
| MCCC 1K01332 | A. macleodii | East Pacific Ocean | Surface | − |
| MCCC 1K01840 | A. macleodii | Western Pacific Ocean | Subsurface (30 m) | − |
| MCCC 1K01294 | A. macleodii | Western Pacific Ocean | Subsurface (75 m) | − |
| MCCC 1A04487 | A. macleodii | Northwestern Pacific Ocean | Bathypelagic (2700 m) | − |
| MCCC 1A07993 | A. macleodii | Southern Atlantic Ocean | Bathypelagic (2147 m) | − |
| MCCC 1A09262 | A. macleodii | Southern Atlantic Ocean | Bathypelagic (3047 m) | − |
| MCCC 1A00323 | A. macleodii | Atlantic Ocean | Bathypelagic (3542 m) | − |
| MCCC 1K02779 | A. macleodii | Atlantic Ocean | Sediment (2577 m) | − |
| MCCC 1K00460 | A. macleodii | Western Pacific Ocean | Surface | − |
| MCCC 1K00811 | A. macleodii | South China Sea | Surface | − |
| MCCC 1K01274 | A. macleodii | Western Pacific Ocean | Subsurface (100 m) | − |
| MCCC 1K01826 | A. macleodii | Western Pacific Ocean | Subsurface (100 m) | − |
| MCCC 1K00767 | A. macleodii | Eastern Pacific Ocean | Mesopelagic (500 m) | − |
| MCCC 1K01276 | A. macleodii | Western Pacific Ocean | Mesopelagic (300 m) | − |
| MCCC 1K00800 | A. macleodii | Eastern Pacific Ocean | Bathypelagic (1000 m) | − |
| MCCC 1A02046 | A. macleodii | Indian Ocean | Bathypelagic (2391 m) | − |
| MCCC 1K02456 | A. macleodii | Northwestern Indian Ocean | Sediment (1818 m) | − |
| MCCC 1K02451 | A. macleodii | Northwestern Indian Ocean | Sediment (2009 m) | − |
| MCCC 1K02444 | A. macleodii | Northwestern Indian Ocean | Sediment (2540 m) | − |
| MCCC 1K01703 | A. macleodii | Atlantic Ocean | Sediment (2781m) | − |
| MCCC 1K01716 | A. macleodii | Atlantic Ocean | Sediment (2781 m) | − |
| MCCC 1K02452 | A. macleodii | Northwestern Indian Ocean | Olivine (3042 m) | − |
| MCCC 1K01358 | A. macleodii | Eastern Pacific Ocean | Surface | − |
| MCCC 1K01832 | A. macleodii | Western Pacific Ocean | Subsurface (30 m) | − |
| MCCC 1K01839 | A. macleodii | Western Pacific Ocean | Surface | − |
| MCCC 1K01823 | A. macleodii | Western Pacific Ocean | Subsurface (75 m) | − |
| MCCC 1K01842 | A. macleodii | Western Pacific Ocean | Subsurface (75 m) | − |
| MCCC 1F01223 | A. macleodii | Xiamen, China | Algae culture | − |
| MCCC 1K00562 | A. macleodii | Eastern Pacific Ocean | Surface | − |
| MCCC 1K01333 | A. macleodii | Pacific Ocean | Surface | − |
| MCCC 1K02752 | A. macleodii | Atlantic Ocean | Surface | − |
| MCCC 1K02757 | A. macleodii | Atlantic Ocean | Surface | − |
| MCCC 1A08375 | A. macleodii | Eastern Pacific Ocean | Subsurface (25 m) | − |
| MCCC 1K01838 | A. macleodii | Western Pacific Ocean | Subsurface (30 m) | − |
| MCCC 1K01203 | A. macleodii | South China Sea | Subsurface (30 m) | − |
| MCCC 1K01646 | A. macleodii | Atlantic Ocean | Subsurface (50 m) | − |
| MCCC 1K01647 | A. macleodii | Atlantic Ocean | Subsurface (50 m) | − |
| MCCC 1K01648 | A. macleodii | Atlantic Ocean | Subsurface (50 m) | − |
| MCCC 1K01684 | A. macleodii | Atlantic Ocean | Subsurface (50 m) | − |
| MCCC 1K01685 | A. macleodii | Atlantic Ocean | Subsurface (50 m) | − |
| MCCC 1K01109 | A. macleodii | South China Sea | Mesopelagic (150 m) | − |
| MCCC 1K00282 | A. macleodii | Eastern Pacific Ocean | Bathypelagic (1000 m) | − |
| MCCC 1K01191 | A. macleodii | South China Sea | Bathypelagic (1000 m) | − |
| MCCC 1K01935 | A. macleodii | South China Sea | Bathypelagic (1262 m) | − |
| MCCC 1K01957 | A. macleodii | Western Pacific Ocean | Bathypelagic (1311 m) | − |
| MCCC 1K01949 | A. macleodii | Western Pacific Ocean | Bathypelagic (1383 m) | − |
| MCCC 1K01725 | A. macleodii | Atlantic Ocean | Sediment (1700 m) | − |
| MCCC 1K02450 | A. macleodii | Northwestern Indian Ocean | Bathypelagic (2540 m) | − |
| MCCC 1K00283 | A. macleodii | Eastern Pacific Ocean | Bathypelagic (3000 m) | − |
| MCCC 1A09138 | A. macleodii | Southern Atlantic Ocean | Bathypelagic (3399 m) | − |
| MCCC 1K00863 | A. macleodii | Southern Pacific Ocean | Bathypelagic (3738 m) | − |
| MCCC 1K00848 | A. macleodii | Southern Pacific Ocean | Bathypelagic (4258 m) | − |
| MCCC 1K00563 | A. macleodii | Eastern Pacific Ocean | Bathypelagic (4860 m) | − |
| MCCC 1K00409 | A. macleodii | Eastern Pacific Ocean | Bathypelagic (4898 m) | − |
| MCCC 1K02449 | A. macleodii | Southwestern Indian Ocean | Bathypelagic (5152 m) | − |
| MCCC 1K01331 | A. macleodii | Pacific Ocean | Bathypelagic (5302 m) | − |
| MCCC 1K00284 | A. macleodii | Eastern Pacific Ocean | Bathypelagic (5089 m) | − |
| MCCC 1K00297 | A. macleodii | Eastern Pacific Ocean | Mesopelagic (300 m) | − |
| MCCC 1K00097 | A. macleodii | Northern Atlantic Ocean | Sediment (2577 m) | − |
| MCCC 1K00561 | A. macleodii | Eastern Pacific Ocean | Surface | − |
| MCCC 1K01132 | A. macleodii | South China Sea | Subsurface (30 m) | − |
| MCCC 1A09719 | A. macleodii | Southern Atlantic Ocean | Subsurface (50 m) | − |
| MCCC 1K00753 | A. macleodii | South China Sea | Mesopelagic (450 m) | − |
| MCCC 1A03444 | A. macleodii | South China Sea | Mesopelagic (812 m) | − |
| MCCC 1K00759 | A. macleodii | South China Sea | Bathypelagic (1000 m) | − |
| MCCC 1K01190 | A. macleodii | South China Sea | Bathypelagic (1000 m) | − |
| MCCC 1K02216 | A. macleodii | Western Pacific Ocean | Bathypelagic (1200 m) | − |
| MCCC 1K01923 | A. macleodii | South China Sea | Bathypelagic (1262 m) | − |
| MCCC 1K02267 | A. macleodii | Western Pacific Ocean | Bathypelagic (1267 m) | − |
| MCCC 1A01084 | A. macleodii | Indian Ocean | Bathypelagic (2488 m) | − |
| MCCC 1A09498 | A. macleodii | Southern Atlantic Ocean | Sediment (1598 m) | − |
| MCCC 1K00565 | A. abrolhosensis | Eastern Pacific Ocean | Bathypelagic (5098 m) | − |
| MCCC 1A09157 | A. abrolhosensis | Southern Atlantic Ocean | Sediment | − |
| MCCC 1A09130 | A. abrolhosensis | Southern Atlantic Ocean | Sediment | − |
| MCCC 1K01033 | A. abrolhosensis | Southern Pacific Ocean | Bathypelagic (5306 m) | − |
| MCCC 1F01092 | A. abrolhosensis | Chiu-lung River | Sediment (0 m) | − |
| MCCC 1K01723 | A. abrolhosensis | Atlantic Ocean | Sediment (1700 m) | − |
| MCCC 1K01726 | A. abrolhosensis | Atlantic Ocean | Sediment (1700 m) | − |
| MCCC 1A09116 | A. abrolhosensis | Southern Atlantic Ocean | Sediment (2596 m) | − |
| MCCC 1K01719 | A. abrolhosensis | Atlantic Ocean | Sediment (2727 m) | − |
| MCCC 1K01720 | A. abrolhosensis | Atlantic Ocean | Sediment (2727 m) | − |
| MCCC 1K01717 | A. abrolhosensis | Atlantic Ocean | Sediment (2781 m) | − |
| MCCC 1K01724 | A. abrolhosensis | Atlantic Ocean | Sediment (3110 m) | − |
| MCCC 1K01727 | A. abrolhosensis | Atlantic Ocean | Sediment (3110 m) | − |
| MCCC 1A08167 | A. abrolhosensis | Southern Atlantic Ocean | Seafloor rocks (2779 m) | − |
| MCCC 1K00364 | A. abrolhosensis | Eastern Pacific Ocean | Sediment (5368 m) | − |
| MCCC 1A07988 | A. mediterranea | Southern Atlantic Ocean | Bathypelagic (5610 m) | − |
| MCCC 1A08050 | A. mediterranea | Southern Atlantic Ocean | Sediment (2481 m) | − |
| MCCC 1A05262 | A. mediterranea | Southwestern Pacific Ocean | Bathypelagic (2695 m) | − |
| MCCC 1F01155 | A. mediterranea | South China Sea | Surface | − |
| MCCC 1K02248 | A. mediterranea | Western Pacific Ocean | Bathypelagic (1100 m) | − |
| MCCC 1K02087 | Alteromonas sp. | South China Sea | Bathypelagic (1700 m) | − |

“+” indicates infected; “−” indicates uninfected;

The superscript letter “T” represents type strain.
